# Effectiveness of rehabilitation training combined with acupuncture on aphasia after cerebral hemorrhage: A systematic review protocol of randomized controlled trial

**DOI:** 10.1097/MD.0000000000016006

**Published:** 2019-06-14

**Authors:** Xin-shu Dong, Guang-fu Song, Cheng-ji Wu, Chun-yin Zou, Guang-tao Sun, Zuo-yi Huang

**Affiliations:** a Rehabilitation Unit of Fourth Department of Neurology; b Brain Surgery Unit of Fourth Department of Neurology; c Fourth Department of Neurology, First Affiliated Hospital of Jiamusi University, Jiamusi, China.

**Keywords:** acupuncture, aphasis, cerebral hemorrhage, effectiveness, rehabilitation training

## Abstract

**Background:**

This study aims to systematically evaluate the effectiveness of rehabilitation training (RT) combined with acupuncture on aphasia after cerebral hemorrhage (CH).

**Methods:**

PUBMED, Cochrane Central Register of Controlled Trials, EMBASE, Web of Science, Ovid, Cumulative Index to Nursing and Allied Health Literature, Allied and Complementary Medicine Database, Chinese Biomedical Literature Database, and China National Knowledge Infrastructure will be searched to identify any potential studies from inception to March 1, 2019, without language restrictions. All randomized controlled trials and case-controlled studies assessing the effectiveness of RT combined with acupuncture for the treatment of aphasia following CH will be included in this study. Cochrane risk of bias tool will be used to determine the methodological quality for included studies. RevMan 5.3 software (Cochrane Community, London, UK) will be utilized to perform statistical analysis.

**Results:**

This study will systematically evaluate the effectiveness of RT and acupuncture for aphasia post CH. Primary outcome includes aphasia, which can be measured by Aachener Aphasia Test or Communicative Activity Log or other related scales. Secondary outcomes consist of speech performance, as assessed by Western Aphasia Battery-Revised; measure of skill in Supported Conversation scales; measure of Participation in Conversation scales; types of strategies used in conversation; occurrence and repair of conversation breakdowns; as well as any adverse events.

**Conclusion:**

The results of this study will provide present evidence on assessing effectiveness of RT and acupuncture after CH.

**Dissemination and ethics:**

The findings of this study are expected to be published in peer-reviewed journals. It does not require ethical approval, because no individual data will be utilized in this study.

**Systematic review registration:**

PROSPERO CRD42019131587.

## 1 Introduction

Cerebral hemorrhage (CH) is a very common disease in the neurosurgery department.^[1–3]^ Its incidence ranges from 50.6 to 80.7 per 100,000 people, which accounts for 18.8% to 47.6% of all acute cerebrovascular diseases among Chinese population.^[4]^ The high rates of mortality and disability, as well as the high financial burden are associated with this disorder.^[5–8]^ In addition, most survivors suffer from server impairments, such as limbs paralysis or weakness, depression, bladder or bowel disorders, and aphasia.^[9–20]^ Therefore, timely and effective managements are very important for the CH survivors.

Many managements are reported to treat CH survivors effectively, such as medications, supportive care, surgery, physical therapy, acupuncture, rehabilitation training (RT), and any other therapies,^[21–24]^ especially for CH survivors with aphasia. Several clinical trials have reported that acupuncture and RT can treat CH survivors with aphasia and have achieved promising effectiveness.^[25–32]^ However, no study systematically assesses the effectiveness of acupuncture and RT for aphasia following CH.

## 2 Methods and analysis

### 2.1 Study registration

This study has already completed registration on PROSPERO (CRD42019131587). It will comply with the preferred reporting items for systematic reviews and meta-analyses (PRISMA) protocol statement guidelines.

### 2.2 Eligibility criteria for study selection

#### 2.2.1 Participants/population

Patients with aphasia after CH will be included without any limitations, such as gender, age, and so on. However, we will exclude studies with aphasia before CH, or caused by any other disorders.

#### 2.2.2 Interventions/exposure

Any types of acupuncture combined RT will be used as only treatment for patients in the experimental group.

The control group can receive any treatments except acupuncture or RT alone or combination.

#### 2.2.3 Study types

All randomized controlled trials (RCTs) and case-controlled studies (CCS) assessing the effectiveness of RT combined with acupuncture for aphasia after CH will be considered for inclusion in this study. However, any other studies except the RCTs or CCSs will not be considered in this study.

#### 2.2.4 Outcome measurements

Primary outcome of aphasia will be measured by Aachener Aphasia Test or Communicative Activity Log or other related scales. Secondary outcomes include speech performance, as assessed by Western Aphasia Battery-Revised; measure of skill in Supported Conversation scales; measure of Participation in Conversation scales; types of strategies used in conversation; occurrence and repair of conversation breakdowns, as well as any adverse events.

### 2.3 Literature search

We will search the following literature sources from their inceptions to the April 1, 2019, without any language restrictions: PubMed, Cochrane Central Register of Controlled Trials, EMBASE, Web of Science, Ovid, Cumulative Index to Nursing and Allied Health Literature, Allied and Complementary Medicine Database, Chinese Biomedical Literature Database, and China National Knowledge Infrastructure. In addition, we will also search Google scholar, clinical registry, reference lists of all relevant reviews and eligible studies, and conference proceedings. All RCTs and CCS on assessing the effectiveness of RT combined with acupuncture for the treatment of aphasia following CH will be considered. The detailed strategy of PubMed is summarized in Table 1. The equivalent search strategies will be utilized to other electronic databases.

**Table 1:**
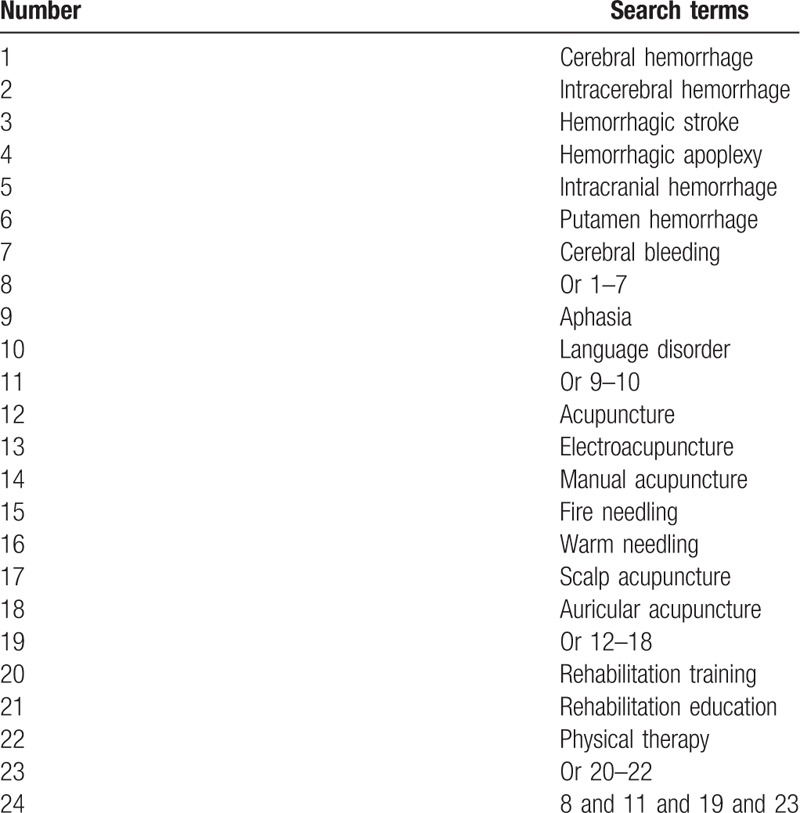
Search strategy for PubMed database.

### 2.4 Study selection

Two authors will independently scrutinize the titles or abstracts initially for all literature records first. Then, full texts will be reviewed carefully based on the predefined eligibility criteria. The results of the study selection in this study will be reported in the flowchart and will follow PRISMA guidelines with detailed reasons of exclusion for each study. A third author will resolve any disagreements between 2 reviewers.

### 2.5 Data extraction and management

Two authors will extract following information from each included study and will save all data in a data extraction sheet: general information (first author, published year, region, age, sex, ethnicity, disease types); relevant study methods (sample size, randomization, allocation, and blinding); interventions methods (details of interventions, including dosage, frequency, treatment duration); and outcome measurements (primary and secondary outcomes, adverse events, and any others). Any divergences between 2 authors will be resolved by a consensus or arbitration with a third author.

### 2.6 Dealing with missing data

The primary authors will be contacted to acquiring the insufficient or missing data if any of them arise. If we are not able to obtain those data, we will just analyze the available data, and also will discuss its potential impact in the Discussion section.

### 2.7 Risk of bias assessment

Cochrane risk of bias tool will be used to evaluate the risk of bias assessment for all eligible studies. It assesses each study through 7 aspects and is classified into 3 levels of low, unclear, and high risk of bias. Two reviewers will independently assess each study, and a third reviewer will involve solving any divergences between 2 reviewers in this study.

### 2.8 Reporting bias

Funnel plot and Egger regression test will be carried out to check if there is any reporting bias when more than 10 studies are entered in this study.

### 2.9 Statistical analysis

RevMan 5.3 software will be applied to conduct statistical analysis. All continuous data will be presented as mean difference or standardized mean difference with 95% confidence intervals. All the dichotomous data will be showed as risk ratio with 95% confidence intervals.

We will use *I*^*2*^ test to check any heterogeneity among included studies. *I*^*2*^ ≤50% means that acceptable heterogeneity is identified, and a fixed-effect model is used for data pooling. Otherwise, it means that high heterogeneity is found, and a random-effect model is used for data pooling. Under this situation, we will also carry out subgroup analysis based on the different types of treatments, control interventions, and outcome measurements. If there is still very high heterogeneity, data will not be pooled, and meta-analysis will not be carried out for reports of outcome results. Instead, we will report all outcome results as narrative summary. Moreover, we will also carry out sensitivity analysis to check robustness and stability of pooled results by removing low-quality studies.

## 3 Discussion

This study will systematically assess the effectiveness and safety of acupuncture combined with RT for the treatment of aphasia following CH. Although several clinical trials have reported that acupuncture combined with RT can effectively treat aphasia after CH, no study has systematically explored this issue. Therefore, in this study, we will first evaluate the effectiveness and safety of acupuncture and RT for the treatment of patients with aphasia after CH. The results of this study will provide most current evidence on assessing effectiveness of acupuncture and RT on aphasia following CH.

## Author contributions

**Conceptualization:** Xin-shu Dong, Guang-fu Song, Cheng-jie Wu, Chun-yin Zou, Guang-tao Sun.

**Data curation:** Xin-shu Dong, Guang-fu Song, Cheng-jie Wu, Chun-yin Zou, Guang-tao Sun, Zuo-yi Huang.

**Formal analysis:** Xin-shu Dong, Guang-fu Song, Cheng-jie Wu, Chun-yin Zou, Guang-tao Sun.

**Funding acquisition:** Xin-shu Dong.

**Investigation:** Cheng-jie Wu, Chun-yin Zou.

**Methodology:** Xin-shu Dong, Guang-fu Song, Guang-tao Sun, Zuo-yi Huang.

**Project administration:** Cheng-jie Wu.

**Resources:** Xin-shu Dong, Chun-yin Zou, Guang-tao Sun, Zuo-yi Huang.

**Software:** Guang-fu Song, Guang-tao Sun.

**Supervision:** Cheng-jie Wu.

**Validation:** Xin-shu Dong, Chun-yin Zou, Zuo-yi Huang.

**Visualization:** Xin-shu Dong, Chun-yin Zou, Guang-tao Sun, Zuo-yi Huang.

**Writing – original draft:** Xin-shu Dong, Guang-fu Song, Cheng-jie Wu, Guang-tao Sun, Zuo-yi Huang.

**Writing – review & editing:** Xin-shu Dong, Guang-fu Song, Cheng-jie Wu, Chun-yin Zou, Guang-tao Sun, Zuo-yi Huang.
