# Effectiveness of rehabilitation training combined with acupuncture on aphasia after cerebral hemorrhage: A systematic review protocol of randomized controlled trial: Erratum

**DOI:** 10.1097/MD.0000000000018599

**Published:** 2019-12-20

In the article, “Effectiveness of rehabilitation training combined with acupuncture on aphasia after cerebral hemorrhage: A systematic review protocol of randomized controlled trial”,^[1]^ which appeared in Volume 98, Issue 24 of *Medicine*, the names of Xin-shu Dong, and Chun-yin Zou should be correctly shown as Shu-xin Dong, and Chun-ying Zou. In the section of Author contributions, the name of Cheng-jie Wu should be correctly shown as Cheng-ji Wu.
